# Meta-QTL and haplo-pheno analysis reveal superior haplotype combinations associated with low grain chalkiness under high temperature in rice

**DOI:** 10.3389/fpls.2023.1133115

**Published:** 2023-03-08

**Authors:** Anita Kumari, Divya Sharma, Priya Sharma, Sahil, Chaoxin Wang, Vibha Verma, Arun Patil, Md Imran, Madan Pal Singh, Kuldeep Kumar, Kumar Paritosh, Doina Caragea, Sanjay Kapoor, Girish Chandel, Anil Grover, S. V. Krishna Jagadish, Surekha Katiyar-Agarwal, Manu Agarwal

**Affiliations:** ^1^ Department of Botany, University of Delhi, Delhi, India; ^2^ Department of Computer Science, Kansas State University, Manhattan, KS, United States; ^3^ Department of Plant Molecular Biology, University of Delhi, New Delhi, India; ^4^ Department of Plant Molecular Biology and Biotechnology, Indira Gandhi Krishi Vishwavidyalaya, Chattisgarh, India; ^5^ Division of Plant Physiology, Indian Council of Agricultural Research (ICAR), New Delhi, India; ^6^ National Institute for Plant Biotechnology, Indian Council of Agricultural Research (ICAR), New Delhi, India; ^7^ Centre for Genetic Manipulation of Crop Plants, New Delhi, India; ^8^ Department of Plant and Soil Science, Texas Tech University, Lubbock, TX, United States

**Keywords:** starch metabolism, grain chalkiness, meta-QTL analysis, haplotype, haplo-pheno analysis, granule bound starch synthase I, starch synthase IIa

## Abstract

Chalk, an undesirable grain quality trait in rice, is primarily formed due to high temperatures during the grain-filling process. Owing to the disordered starch granule structure, air spaces and low amylose content, chalky grains are easily breakable during milling thereby lowering head rice recovery and its market price. Availability of multiple QTLs associated with grain chalkiness and associated attributes, provided us an opportunity to perform a meta-analysis and identify candidate genes and their alleles contributing to enhanced grain quality. From the 403 previously reported QTLs, 64 Meta-QTLs encompassing 5262 non-redundant genes were identified. MQTL analysis reduced the genetic and physical intervals and nearly 73% meta-QTLs were narrower than 5cM and 2Mb, revealing the hotspot genomic regions. By investigating expression profiles of 5262 genes in previously published datasets, 49 candidate genes were shortlisted on the basis of their differential regulation in at least two of the datasets. We identified non-synonymous allelic variations and haplotypes in 39 candidate genes across the 3K rice genome panel. Further, we phenotyped a subset panel of 60 rice accessions by exposing them to high temperature stress under natural field conditions over two Rabi cropping seasons. Haplo-pheno analysis uncovered haplotype combinations of two starch synthesis genes, *GBSSI* and *SSIIa*, significantly contributing towards the formation of grain chalk in rice. We, therefore, report not only markers and pre-breeding material, but also propose superior haplotype combinations which can be introduced using either marker-assisted breeding or CRISPR-Cas based prime editing to generate elite rice varieties with low grain chalkiness and high HRY traits.

## 1 Highlights

A combination of natural variations in starch metabolism genes SSIIa and GBSSI is associated with low grain chalkiness and high head yield under high temperature in rice.

## 2 Introduction

Rice (*Oryza sativa* L.), a primary staple food, is life for about half of the world’s population. Besides providing food security, rice farming generates secure livelihood for millions across the world (Le, 2016). Asia is a major producer as well as consumer of rice accounting for over 90% of world’s production and consumption (www.fao.org; Hossain et al., 2006; Muthayya et al., 2014). Asia (~4.67 billion) followed by Africa (~1.37 billion) are the two most populous continents and even though the population growth rate in Africa is nearly 3 times (2.45%) more than Asia (0.83%), the absolute population increase in Asia far exceeds that of Africa. Notwithstanding that surplus rice is being produced and consumption has been declining in Asia, it is predicted that the exponential increase in population will soon outstrip the incremental gains in rice production. The predicted gap in demand and supply is likely to be further widened by the climate change-accompanied abiotic factors that adversely affect the yield and quality of rice grains. Predictive modeling by the Intergovernmental Panel on Climate Change [IPCC, Sixth Assessment Report, 2021 (www.ipcc.ch)] estimates that even if all man-made activities are ceased, global temperature will continue to rise by 1.5°C to 2°C in the next two decades, which would be catastrophic to agriculture globally, including rice cultivation. Although rice is primarily cultivated in humid tropical and subtropical climates, minor increase in growth temperatures, especially high night-time temperatures, results in reduced spikelet fertility and sub-optimum grain characteristics. This includes, increased chalky grain ratio which causes significant reduction in grain yield and inferior quality attributes (Lyman et al., 2013; Wu et al., 2016).

Rice grains, in their journey from fields to the consumer, are scrutinized on several interrelated grain quality characteristics such as appearance, cooking, palatability and nutritional value. While the intermediaries, such as millers, are primarily interested in higher recovery of intact grain (also known as head rice yield or HRY), the consumer acceptance depends on the flavor, appearance and cooking quality (He et al., 1999). Grain chalk, an opaque and disordered area in the endosperm due to increased air spaces between the malformed starch granules (Lisle et al., 2000) is a highly unacceptable trait that accounts for many inferior grain characteristics. Based on the position of chalk deposition in rice grain, chalkiness is categorized as white-back, white-base, white-belly, white-core and milky-white (Tashiro and Wardlaw, 1991). The degree of chalkiness influences the recovery of head rice and since rice is preferably consumed as an intact whole kernel head rice fetches 40-50% higher price than the broken grains (Cooper et al., 2008; Chen et al., 2011; Zhao and Fitzgerald, 2013; Sreenivasulu et al., 2015). Consequently, besides boosting rice production, improvement of rice grain milling quality, with respect to high HRY and low grain chalkiness, are the key challenges in rice improvement programs worldwide (Nelson et al., 2011; Zhao and Fitzgerald, 2013).

Rice endosperm starch is composed of two main components: amylose and branched chain amylopectin. While granule-bound starch synthase I (*GBSSI*/Waxy) elongates amylose, soluble starch synthases (SSs), starch branching enzymes (BEs) and starch debranching enzymes (DEBs) are involved in synthesis and branching of amylopectin (Smith et al., 1997). Perturbations in the starch metabolism pathway leads to an aberrant amylose-amylopectin composition and altered starch structure, culminating in formation of chalk (Butardo et al., 2011). Both the expression and activity of starch metabolism genes and proteins, respectively, are modulated by high temperatures for e.g., starch synthesis genes are downregulated, while genes for starch degrading enzymes are upregulated by heat stress (Yamakawa et al., 2007; Liu et al., 2010b; Yamakawa and Hakata, 2010; Lin et al., 2017). Functional analysis studies have identified several genes like pyruvate orthophosphate dikinase (*PPDK*), starch synthase IIIa (*SSIIIa*), UDP-glucose pyrophosphorylase (*UGPase 1*) and cell-wall invertase, associated with chalk formation in rice (Kang et al., 2005; Fujita et al., 2007; Wang et al., 2008; Woo et al., 2008). However, in view of the spatial and temporal complexity of starch synthesis and deposition in developing rice grains, chalkiness appears to be a complex quantitative trait with polygenic inheritance.

Several research groups have mapped quantitative trait loci (QTL) for grain chalkiness and its associated traits (**Table 1**). Few chalk-associated QTLs have been fine mapped such as Chalk5 that codes for a vacuolar H^+^-translocating pyrophosphatase and whose enhanced expression contributes to increased chalkiness (Li et al., 2014). Similarly, non-synonymous variations in OsAGPS1 gene within the qACE9 QTL affected chalk accumulation (Gao et al., 2016a). Takehara et al. (2018) dissected the Apq1 QTL to three genes and based on their temporal expression identified OsSUS3 as the candidate gene. However, the majority of QTLs from independent studies have not yet been fine mapped and have overlapping genomic regions owing to variable experimental designs and environment, genetic background of parents, population size, type and density of molecular markers and the statistical approach utilized for downstream analysis. Traditionally, the fine mapping of QTL to mine the causal gene/(s) involves mapping in the advanced generation (e.g., RILs) with an increased number of markers to obtain a condition where chances of recombination between the markers and traits are subtle. An alternative approach that can help in fine mapping, is meta-analysis of QTLs (MQTL) that are identified from multiple studies. MQTL analysis is fast emerging as an effective approach for reducing the confidence intervals (CI) of overlapping QTLs so that promising markers and genes associated with the concern trait could be rapidly mined.

**Table 1:** Summary of the grain chalkiness QTL mapping studies in rice used to perform the meta-analysis.

Mapping Population			Markers Used		QTL identified		Reference
Parents	Type	Size	Type	Number	Trait^*^	Number	
Sasanishiki x Habataki	ILs	37	SSR	142	PGWC	6	Bian et al., 2013
Pusa1266 X Jaya	RILs	310	SSR	116	PGWC, CS	2	Chandusingh et al., 2013
PYZXXP02430	RIL	192	SNP	2711	PGWC, DC	26	Chen et al., 2016
Chunjiang X Taichung	DH	116	SSR,STS	188, 19	CK, CS CD	10	Dai et al., 2016
XieqingzaoXMilyang	RIL	209	SSR	240	PGWC, DC,ET	40	Mei et al., 2013
KBNT lpa X Zhe733	RIL	187	SSR, SNP	174, 1	Chalk	10	Edwards et al., 2017
GSOR301227XGSOR 301190	RIL	276	SSR	132	Chalk	3	Eizenga et al., 2019
G46BXK1075	RIL	182	SSR	33	PGWC	3	Gao et al., 2016a
9311XPA64s	RIL	104	SNP	4	ACE, DC, PGWC	19	Gao et al., 2016b
AsominoriXIR-24	BC	9711	SSR	10	PGWC	1	Guo et al., 2011
AsominoriXIR-24	RIL, CSSL	7,166	RFLP	375	PGWC	10	Chen et al., 2011
CypressXPanda	RIL	137	AFLP, SSR	53,235	KCAb	3	Kepiro et al., 2008
Ali-Kazemi X Kadous	RIL	157	SSR	300	Chalk	2	Bazrkar-Khatibani et al., 2019
HanaechizanXNiigatawase	F2, F3	180	SSR	407	WBK	3	Kobayashi et al., 2007
HanaechizanXNiigatawase	RIL	178	SSR	175	WBK	4	Kobayashi et al., 2013
V20AXO.glaberrima	BC	308	RFLP, SSR	110, 20	WC, WCA	2	Li et al., 2004
KoshihikariasXGuchao 2	BC	71	SSR	144	PGWC, DPGWC	58	Wang et al., 2016
AsominoriXIR24	CSSL	66	RFLP	116	PGWC, ACE	15	Liu et al., 2010a
KoshihikarixXC602	RIL	261	SSR	236	PGWC	3	Liu et al., 2012
Xiushui79XC Bao	RIL	254	SSR	111	CGR, CD	15	Liu et al., 2013
N22X Nanjing35	F2, BILs	306	SSR	369	PGWC	10	Lu et al., 2013
TeqingXIR BB	BC	190	SSR	11	PGWC, DC	2	Mei et al., 2018
TsukushiromanXChikushi52	RIL	88	SSR, CAPS, dCAPS	70, 4, 2	MW	4	Miyahara et al., 2017
KoshihikariXYamadanishiki	RIL	284	SNP	703	WCE	6	Okada et al., 2017
ZS97XH94	DH	–	SSR	218	WBR, WBA, WCR, CA,CR	70	Peng et al., 2014a
V20BXYVB	BC	100	SNP	694	CR, CA	3	Peng et al., 2016
SamgangXNagdong	DH	120	SSR, STS	56, 116	PWC WCA, WBA, PWB, GCA, PGWC	16	Qin et al., 2009
KoshijiwaseXChiyonishiki	RIL	107	SSR	106	WK	4	Tabata et al., 2007
Zhenshan97XMinghui63	RIL	479	RFLP, SSR	166, 5	Chalk, WB, WC	9	Tan et al., 2000
HabatakiXSasanishiki	CSSLs	39	SSR	23	BW, MW, WBB	6	Terao and Hirose, 2018
TsukushiromanXChikushi52	RIL	88	SSR, SNP	71, 6	WB, BW	9	Wada et al., 2015
AsominoriXIR-24	RIL, CSSL	66	RFLP	116	PGWC, CA, DC	11	Wan et al., 2005
KoshihikariXNona Bokara	CSSLs	154	CAPs, SSR	102	PCRG, DC	2	Hao et al., 2009
O. sativaXO. rufipogon	IL	121	SSR	124	CR	1	Yuan et al., 2010
Beilu130XJin23B	RIL	184	SSR	190	WCR, WBR	10	Yun et al., 2016
KoshihikariXKasalath	BC	182	RFLP	162	PGWC	3	Zheng et al., 2012
9311XPA64s	BC	3221	SSR	202	PGWC	1	Zhou et al., 2009
XBXZhonghui	BC4F4	–	SSR	25	PGWC	1	Zhu et al., 2018

^*^PGWC, Percentage Grain with Chalkiness; DPGWC, Degree of Percentage Grain with Chalkiness; DC, Degree of Endosperm Chalkiness; ET, Endosperm Transparency; CA, Chalkiness Area; WBe, White Belly; WBa, White Back; WBR, White Back Rate; WBA, White Back Area; WC, White core; WCR, White Core Rate; WCA, White Core Area; MW, Milky White; BW, Basal White; WBB, White Back and Basal

We performed a Meta-QTL analysis of the QTLs reported for grain chalkiness in rice to discover the meta-regions associated with it. The identified MQTLs encompassed 5262 genes, which were further shortlisted to 49 based on their differential expression patterns in chalk-associated expression datasets. Further, we performed a haplo-pheno analysis for two of the most important starch synthesis genes and identified haplotype combinations that contribute to low grain chalk under high temperature stress. Our study highlights the relationship between high temperatures and rice grain chalk formation, by establishing the (1) relevance of a QTL meta-analysis in the identification of consensus and precise QTLs associated with grain chalkiness, (2) role and importance of differentially regulated genes from the identified meta-QTL genes, (3) identification of common cis-regulating elements in the candidate genes, (4) discovery of haplotypic variations in the candidate genes across the 3K rice genome panel, (5) haplo-pheno analysis of promising candidate genes in a subset of the 3K panel, and (6) association of haplo-pheno data with the starch granules morphology in the rice endosperm. Our work identifies superior allelic combinations which can be readily employed for generation of climate-resilient rice varieties.

## 3 Material and methods

### 3.1 Literature survey, QTL data collection, and input file preparation

Survey of literature published till 2021 on QTLs contributing towards rice grain chalkiness was performed resulting in the identification of 403 QTLs encompassing several chalk related traits. A total of 38 independent mapping studies (compiled as **Table 1**) involving 30 biparental rice populations were included in the MQTL analysis. QTL data constituting name, trait, chromosome, log of odds (LOD) score, phenotypic variance (R^2^) value, position and confidence intervals (CI) were compiled in the text format and used as QTL input file. For studies having missing data on LOD score and R^2^ we assumed them to be 2.5 and 10%, respectively, as previously suggested by Khahani et al. (2019). A genetic map file containing information about the genetic distances of markers on each linkage group was organized for each study. To incorporate QTLs derived from the studies based on SNP markers, the physical position of the flanking markers was ascertained on the rice genome and closest markers on the reference map were used.

### 3.2 QTL projection on consensus map and meta-analysis of QTLs

Prior to the meta-analysis, a consensus map was built by integrating the genetic map information of all 38 studies listed in **Table 1** with a rice reference map (Temnykh et al., 2001) using BioMercator v4.2.3 (Arcade et al., 2004; Sosnowski et al., 2012). QTLs having variable CI were then projected onto the consensus map and the meta-analysis of only the projected QTLs was performed by merging all traits as rice grain chalk. We applied the two-step algorithm developed by Veyrieras (Veyrieras 2007), wherein first, the best model, having the lowest Akaike Information Criterion (AIC) value, corresponding to the estimated number of MQTLs was computed. In the second step, appropriate parameters for the meta-analysis were set, including the number of MQTLs to be mapped, following which the MQTLs were generated.

### 3.3 Identification of candidate genes within the meta-QTL regions

For determining the genes underlying the identified MQTLs for rice grain chalkiness, the physical locations of confidence interval (CI) flanking markers were retrieved from the Gramene Marker Database (http://archive.gramene.org/qtl/) (Tello-Ruiz et al., 2022). In case the physical position of a particular marker was not available, the position of the adjacent genetic marker was utilized for identifying the genomic coordinates. The locus IDs of all genes spanning the marker intervals of respective MQTL regions were batch downloaded from the Rice Annotation Project Database (RAP) (Sakai et al., 2013). The obtained genes were then compared with the rice grain chalkiness expression datasets from five microarray-based studies (Yamakawa et al., 2007; Yamakawa and Hakata, 2010; Liu et al., 2010b; Ishimaru et al., 2019; Bakku et al., 2020) and one RNA-seq study (Lin et al., 2017). Genes common between at least two expression studies and the current analysis were considered as candidate genes regulating rice grain chalk. Gene ontology information of these chalk associated 49 candidate genes was collected from ShinyGO 0.76 database (Ge et al., 2020).

### 3.4 *In silico* promoter analysis and expression profile

The promoter sequence comprising 1.5 kb upstream from the translational start site (ATG) was downloaded from the Rice Genome Annotation Project Database (http://rice.uga.edu/) for the identified fundamental set of chalk associated candidate genes. All sequences were analyzed for motifs and regulatory elements on the PlantCare database (Lescot et al., 2002). To unravel the expression pattern of the candidate genes in rice endosperms we retrieved their expression data using Genevestigator (Hruz et al., 2008).

### 3.5 Haplotype analysis

An inbuilt tool of SNP seek database (Mansueto et al., 2016) was used to conduct haplotype analysis of the candidate genes in the 3k rice genome panel using Nipponbare as the reference genome. The number of haplotypes were discovered using the default parameters with calinski criterion (Calinski and Harabasz 1974) for determining the ‘k’ group. Only non-synonymous SNPs were considered while carrying out the analysis.

### 3.6 Plant materials and growth conditions

A subset of sixty rice genotypes from the 3K rice genome panel were chosen for grain chalk phenotyping based on the availability of seeds and uniformity in their phenology. Two independent experiments were conducted during Rabi 2020 and 2021 at IGKV, Raipur (21.2514°N, 81.6296°E). Weather data was recorded on a daily basis and plants were exposed to temperatures between 38-41°C during their seed filling stages. In each year four replicates of 15 plants each were grown in two independent fields and seeds were pooled before assessing grain chalk scores. Plants in both years were grown in open fields under fully flooded conditions to study the effect of high temperature stress coinciding with panicle initiation till physiological maturity.

### 3.7 Rice grain chalk phenotyping

Rice grains harvested at physiological maturity were dehusked using the automatic rice husker TR-250 (Kett, USA) and polished before the chalk imaging analysis. The polished grains were arranged on a transparent sheet kept on the scanner surface in three replicates each containing a non-redundant set of 50 randomly selected seeds. Seeds were scanned at a resolution of 800 dots per inch (dpi) using an Epson Perfection V850 Pro photo scanner. Images were saved in TIFF (.tif) file format for further image analysis. Further image processing and chalk score estimation was performed with Gradient-weighted Class Activation Mapping (Grad-CAM) tool and according to the methodology described by Wang et al. (2022).

### 3.8 Haplotypic and phenotypic correlation analysis

We phenotyped a subset of sixty rice genotypes from the 3K rice panel and categorized them as high chalk, intermediate chalk and low chalk to find out a correlation between the chalk score phenotype and the allelic variation in genotypes with respect to the identified candidate genes. Significance test of the means of haplotypic groups and Tukey’s *post hoc* analyses was computed in R.

### 3.9 Scanning electron microscopic observation of starch granules

Scanning electron microscopy (SEM) of rice grains was performed to validate the chalk score values as determined by the image analysis. Mature and dry seeds were transversely fractured by putting pressure in the center of the seed by a surgical blade and were coated with 0.1 nm Chromium using Quorum Q150V plus Sputter coater (Peng et al., 2014b). The coated seeds were visualized under 5 keV electron beam energy using TESCAN Clara Scanning Electron Microscope (Tescan Orsay Holding, Czech Republic).

## 4 Results

### 4.1 Grain chalk QTLs in rice

**Figure 1:**
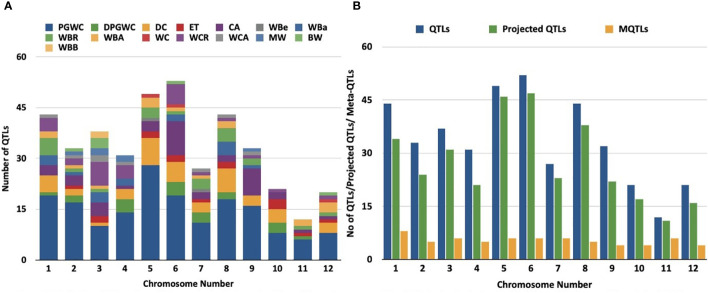
Distribution of QTLs and meta-QTLs associated with rice grain chalk on different chromosomes of rice. **(A)** Trait-wise distribution of initial QTLs used for the meta-QTL analysis. (PGWC, Percentage Grain with Chalkiness; DPGWC, Degree of Percentage Grain with Chalkiness; DC, Degree of Endosperm Chalkiness; ET, Endosperm Transparency; CA, Chalkiness Area; WBe, White Belly; WBa, White Back; WBR, ; WBA, White Back Area; WC, White core; WCR, White core rate; WCA, White core area; MW, Milky white; BW, Basal White; WBB, White Back and Basal) **(B)** The distribution of QTLs, projected QTLs and meta-QTLs on twelve rice chromosomes.

Studies on discovery of QTLs associated with grain chalk in rice have used varied attributes for quantifying phenotypic variations. However, the maximum number of studies employed “percentage of grains with chalkiness” (PGWC) as a measurement of trait. Grain chalk has also been accredited to other quantifiable attributes such as degree of percentage grains with chalkiness (DPGWC), degree of endosperm chalkiness (DC), endosperm transparency (ET), chalkiness area (CA), white belly (WBe), white back (WBa), white back rate (WBR), white back area (WBA), white core (WC), white core rate (WCR), white core area (WCA), milky white (MW), basal white (BW) and white back and basal (WBB) (**Figure 1A**). We, therefore, collated information on 403 QTLs for all these attributes from 38 different mapping studies (see **Table 1** for references) to perform a MQTL analysis for the grain chalkiness trait in rice. These QTLs were discovered from 30 biparental crosses with a population size ranging from 37 to 3221 lines. Diverse molecular markers used in these studies included 4035 Simple Sequence Repeats (SSRs), 3416 Single Nucleotide Polymorphisms (SNPs), 1045 Restriction Fragment Length Polymorphisms (RFLPs), 532 Amplified Fragment Length Polymorphisms (AFLPs), 4 Cleaved Amplified Polymorphisms (CAPs), 2 Derived Cleaved Amplified Polymorphisms (dCAPs) and 135 Sequence Tagged Sites (STSs) (**Table 1**). The number of QTLs varied between 1 to 70 per population, with chromosome 6 having the highest number of QTLs (52) and chromosome 11 having the lowest number of QTLs (12). Confidence intervals (CI) of QTLs varied from 0.52 to 95.8 cM and the phenotypic variance ranged from 0.09% (Li et al., 2004) to 87.2% (Tan et al., 2000).

### 4.2 Distribution of grain chalk meta-QTLs on the rice genome

**Figure 2:**
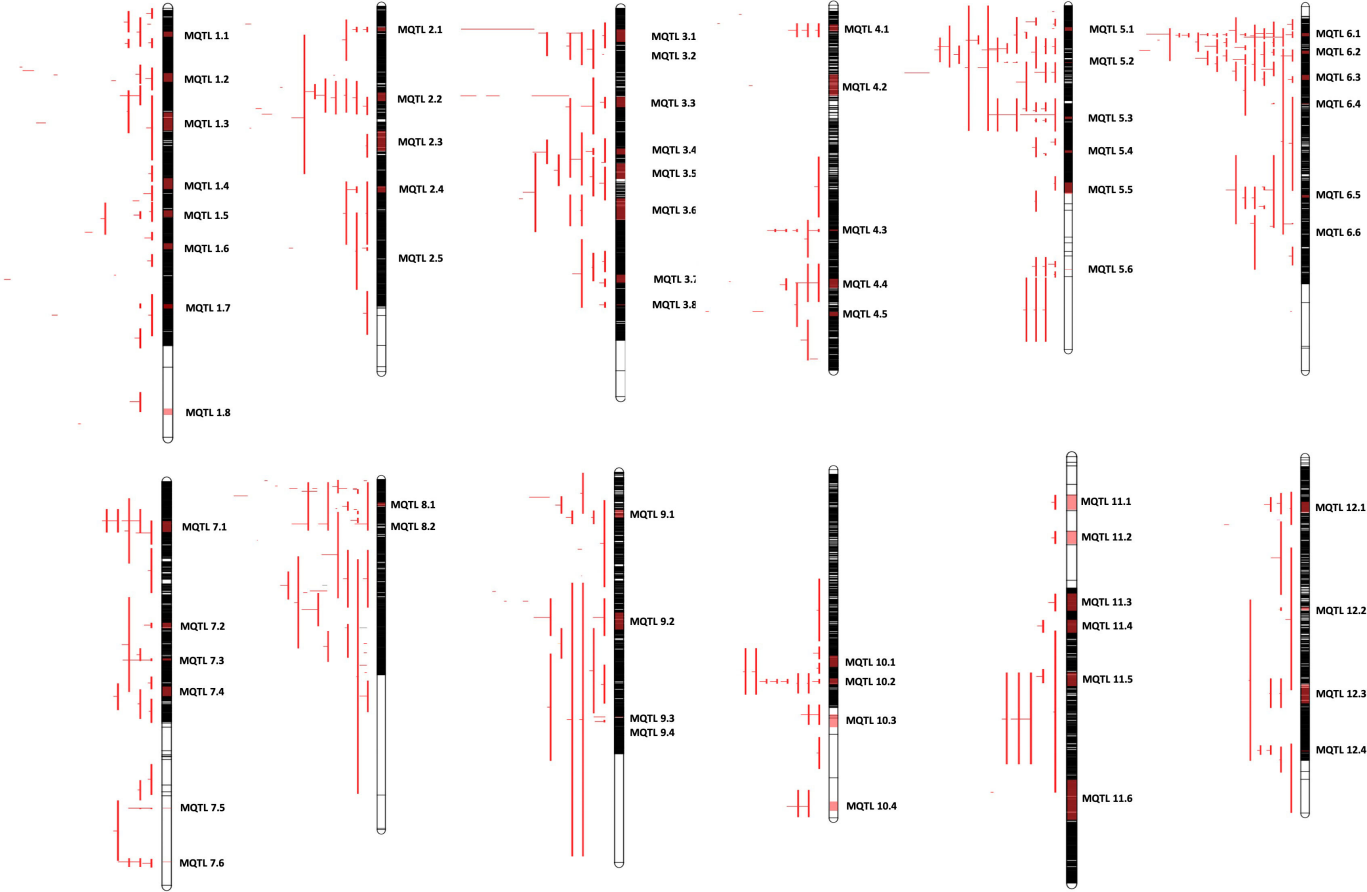
Distribution of meta-QTLs on rice chromosomes. Rice chromosomes are represented by the vertical bars. The names of consensus markers along with their position (cM) are present on the right of the chromosomes. QTLs mapped in various studies are highlighted in different colours on the left of the chromosomes. Coloured blocks on the chromosomes represent the identified meta-QTLs. Vertical bars on the left side of the chromosome represent the confidence interval (CI) of QTL and horizontal bars represent the phenotypic variance.

**Figure 3:**
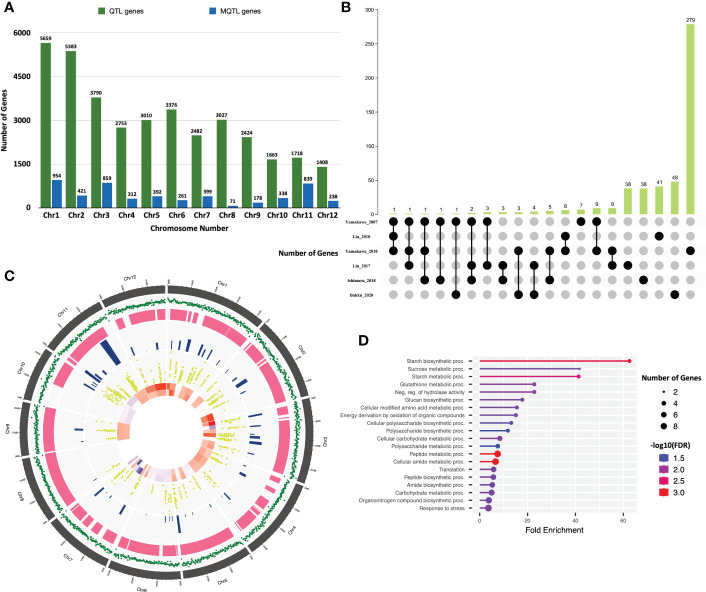
Candidate genes underlying the meta-QTL regions. **(A)** Chromosome-wise distribution of genes underlying QTLs and the identified meta-QTLs in rice. **(B)** Upset plot highlighting the number of differentially regulated genes identified in six rice grain chalk-related transcriptomic studies. **(C)** Distribution pattern of the gene density, QTLs, meta-QTLs, meta-QTL spanning genes and candidate genes identified on the rice genome. The outermost circle represents the chromosomes position on the rice genome in Mb. The second circle with green color outlines the gene density on the rice genome. The third and fourth inner circles display the number of initial chalk QTLs and identified meta-QTLs, respectively. The fifth circle represents the MQTL underlying genes., while the innermost circle represents heatmap of the identified candidate genes (red represents upregulation, while blue signifies downregulation). **(D)** Gene Ontology enrichment analysis of 49 candidate genes representing the biological process. Created using ShinyCircos Yu et al., 2018.

We next generated a consensus map from the 38 QTL maps and the rice reference map (Temnykh et al., 2001). The consensus map consisted of 11,141 markers with a genetic length of 2052.37cM. From the 403 initially retrieved QTLs, 330 (82%) were successfully projected on the consensus maps (**Figure 1B**). Meta-analysis of the 330 projected QTLs identified 64 MQTLs (**Table 2**, **Figure 2**; **Supplementary Figure 1**) on the basis of Veyrieras algorithm of BioMercator v4.2.3. The best MQTL model for each chromosome was selected on the basis of values of AIC, AIC correction (AICc), AIC 3 candidate (AIC3), Bayesian information criterion (BIC), and Average Weight of Evidence (AWE) for each chromosome. The 64 MQTLs were randomly scattered across all the twelve rice chromosomes and their number varied from 2 on Chromosome 8 to 8 on each Chromosomes 1 and 3 (**Figure 2**). Going strictly by definition, only MQTLs identified from at least 2 overlapping QTLs clustered on a consensus map are considered for the downstream analysis, while MQTLs derived from a single QTL are omitted (Yang et al., 2019; Kumar and Nadarajah, 2020). Therefore, MQTLs 1.6, 1.8, 4.2, 4.5, 11.1, 11.2, and 11.3 which were derived from a single QTL were not considered for further analysis (**Table 2**). Clustering of multiple QTLs identified from independent mapping populations that were phenotyped in different environments on the consensus map signifies the importance of the MQTLs identified. We, therefore, used a cut off of 10 QTLs clustering together to predict the most significant MQTLs. MQTL 6.1 derived from 14 overlapping QTLs (mean R^2^ value of 23.7%), and MQTL 6.2 derived from 11 QTLs (mean R^2^ value of 19.6%) indicated that short arm of Chromosome 6 plays an important role in formation of grain chalk. Similarly, MQTL 5.2 was derived due to clustering of 12 QTLs (mean R^2^ value of 18%) suggesting that this genomic region of Chromosome 5 is also important for grain chalkiness in rice. The CI (95% confidence) of the MQTLs ranged from 0.03 to 15.49 cM with a median value of 2.81 cM. Similarly, the physical positions were also reduced and varied from 214 bp to 4.07 Mb with a median value of 0.65 Mb. Notably, the CI of 48 MQTLs (73%) was less than 5 cM, and 52 MQTLs (94%) exhibited a physical distance narrower than 2 Mb. Overall, the meta-analysis resulted in significant reductions in the CIs and physical distance of initial QTLs thereby minimizing the number of candidate genes for further analysis **Figure 3A**.

**Table 2:** Summary of the detected meta-QTLs for grain chalkiness in rice.

Sl No.	MQTL	Chr	QTL Cluster	Position (cM)	Left Marker Name	Position (bp)	Right Marker Name	Position (bp)	CI (95%) (cM)	Physical Interval (bp)	No. of genes
1	MQTL 1.1	1	2	13.59	RM10137	2740418	RM10167	3393293	2.62	652875	121
2	MQTL 1.2	1	4	35.85	RM10513	8072500	RM10578	9187636	4.43	1115136	133
3	MQTL 1.3	1	3	58.68	RM6073	13540292	RM10930	15546516	9.54	2006224	174
4	MQTL 1.4	1	2	91.07	RM11249	22796381	RM11323	24153000	5.69	1356619	162
5	MQTL 1.5	1	3	106.49	RM11408	25854706	RM11448	26814864	3.51	960158	115
6	MQTL 1.6	1	0	123.11	RM1152	30092742	RM11655	30957321	3.25	864579	156
7	MQTL 1.7	1	2	154.27	RM11969	38293480	RM12007	38874652	2.25	581172	93
8	MQTL 1.8	1	1	208.67	RG236	–	RZ538	–	3.63	–	–
9	MQTL 2.1	2	3	11.41	RM12456	2606829	RM12491	3091886	1.99	485057	72
10	MQTL 2.2	2	7	43.72	RM12983	10452238	RM13024	11463387	4.1	1011149	81
11	MQTL 2.3	2	3	64.63	RM13148	14821540	RM13231	17237626	9.68	2416086	174
12	MQTL 2.4	2	3	88.09	RM13456	21613848	RM3688	22395018	3.13	781170	82
13	MQTL 2.5	2	3	116.36	RM13844	29026616	RM13854	29103298	0.26	76682	12
14	MQTL3.1	3	5	12.25	RM14367	2271956	RM14433	3450953	4.83	1178997	193
15	MQTL3.2	3	5	20.19	RM14526	5056073	RM14530	5145876	0.2	89803	15
16	MQTL3.3	3	3	41.03	RM14757	9684987	RM14806	10590987	4.18	906000	153
17	MQTL3.4	3	9	62.7	RM15090	15508298	RM15158	16134623	2.8	626325	67
18	MQTL3.5	3	7	71.38	RM15209	17047128	RM15286	18767961	6.94	1720833	142
19	MQTL3.6	3	5	87.89	RM15326	20800545	RM15422	22682051	7.85	1881506	172
20	MQTL3.7	3	3	117.95	RM15826	29274076	RM15874	30052220	3.41	778144	94
21	MQTL3.8	3	2	129.42	RM16017	32519219	RM16024	32630844	0.39	111625	23
22	MQTL 4.1	4	3	9.22	RM16318	1384306	RM2811	2082855	2.82	698549	42
23	MQTL 4.2	4	1	31.91	RM7472	7090660	RM16556	8865320	8.57	1774660	98
24	MQTL 4.3	4	5	89.22	RM17017	21500378	RM17030	21627469	0.55	127091	12
25	MQTL 4.4	4	5	110.28	RM17279	26378417	RM17312	27317909	3.84	939492	127
26	MQTL 4.5	4	1	122.34	RM17401	29617932	RM17417	29860407	1.92	242475	33
27	MQTL 5.1	5	5	15.07	RM17950	3551113	RM17969	4139221	2.37	588108	51
28	MQTL 5.2	5	12	36.18	RM18185	9063039	RM18189	9163390	0.55	100351	8
29	MQTL 5.3	5	7	71.33	RM6024	17752248	RM7386	18123723	1.55	371475	43
30	MQTL 5.4	5	2	92.78	RM18843	23077962	RM18872	23435326	1.5	357364	45
31	MQTL 5.5	5	2	116.04	RM19132	28241421	RM19224	29722687	7.43	1481266	245
32	MQTL 5.6	5	2	167.19	RG119	–	RG346	–	0.46	–	–
33	MQTL 6.1	6	14	13.31	RM19339	1750988	RM4923	2174616	1.45	423628	73
34	MQTL 6.2	6	11	21.17	RM5754	3762187	RM19477	4117576	1.44	355389	73
35	MQTL 6.3	6	7	32.88	RM19650	6555494	RM5531	7177097	2.46	621603	88
36	MQTL 6.4	6	4	45.06	RM527	9862309	RM527	9862523	0.52	214	1
37	MQTL 6.5	6	5	87.87	RM20215	20867990	RM20235	21162365	1.33	294375	26
38	MQTL 6.6	6	3	100.59	RM20365	–	RM20366	24196636	0.03	–	–
39	MQTL 7.1	7	5	22.52	RM21134	4946994	RM5672	6380197	5.58	1433203	145
40	MQTL 7.2	7	2	70.77	RM445	17462276	RM418	18132520	2.68	670244	48
41	MQTL 7.3	7	3	87.62	RM336	21871205	RM6403	22170728	1.19	299523	37
42	MQTL 7.4	7	4	103.36	RM21963	25338527	RM22046	26605775	4.88	1267248	169
43	MQTL 7.5	7	3	160.61	7042	–	RM1361	–	0.51	–	–
44	MQTL 7.6	7	3	187.12	7042	–	RM1361	–	0.4	–	–
45	MQTL 8.1	8	4	14.86	RM22432	3494017	RM22461	3979625	2.11	485608	58
46	MQTL 8.2	8	4	21.16	RM22543	5271763	RM22546	5345485	0.16	73722	13
47	MQTL 9.1	9	5	13.41	RM23753	3180304	RM2855	3839123	2.64	658819	36
48	MQTL 9.2	9	6	47.75	RM24116	11417353	RM24198	12782400	5.54	1365047	120
49	MQTL 9.3	9	4	78.3	RM24642	19929206	RM3787	20043308	0.35	114102	22
50	MQTL 9.4	9	4	79.66	RM24662	–	RM24663	20318284	0.03	–	–
51	MQTL 10.1	10	4	74.54	RM25607	18067635	RM1108	19161913	4.32	1094278	173
52	MQTL 10.2	10	8	82.45	RM25779	20841416	RM5666	21407562	2.4	566146	81
53	MQTL 10.3	10	2	97.81	RZ421	22493127	RM25934	23075402	5.03	582275	84
54	MQTL 10.4	10	2	130.87	RM18A	–	RG561	–	3.72	–	–
55	MQTL 11.1	11	1	17.74	G1465	–	C6	–	5.76	–	–
56	MQTL 11.2	11	1	31.41	C1003A	–	C1172	–	4.92	–	–
57	MQTL 11.3	11	1	56.38	RM25983	657434	RM26068	2352559	6.82	1695125	256
58	MQTL 11.4	11	2	65.39	RM26127	3086024	RM26194	4372482	5.04	1286458	122
59	MQTL 11.5	11	5	85.78	RM26370	8248744	RM26434	9636542	5.33	1387798	78
60	MQTL 11.6	11	2	132.1	RM26824	18660295	RM27036	22740054	15.49	4079759	383
61	MQTL 12.1	12	3	18.79	RM27580	3327260	RM27657	4413960	4.02	1086700	124
62	MQTL 12.2	12	3	57.05	RM7102	13211325	RM28014	13507963	1.21	296638	12
63	MQTL 12.3	12	4	88.85	RM28331	20694607	RM7018	22163120	7.18	1468513	96
64	MQTL 12.4	12	5	110.31	RM28766	26583689	RM28771	26642770	0.36	59081	6

### 4.3 Candidate gene mining within meta-QTL regions

Since the majority of QTL regions from which MQTLs were derived have not yet been fine mapped, we considered all the genes within MQTLs as putative candidate genes. IDs of the genes for each MQTL were batch retrieved from the Rice Annotation Project Database (RAP), and a total of 5262 non-redundant genes were present within the 64 MQTL regions (**Supplementary Table 1**). Maximum number of genes were in the MQTL 11.6 (383) followed by MQTL 11.3 (256) and MQTL 5.5 (245), while the lowest number of genes were found within MQTLs 6.4 (1) and 12.4 (6) (**Table 2**). A common approach for further shortlisting the putative candidates is to ascertain the differentially expressed (DEGs) MQTL genes for the studied trait (Kong et al., 2020; Mirdar Mansuri et al., 2020; Raza et al., 2020; Khahani et al., 2021). Therefore, we overlapped all MQTL candidate genes with the 6 previously published expression datasets for grain chalkiness in rice (Yamakawa et al., 2007; Yamakawa and Hakata, 2010; Liu et al., 2010b; Lin et al., 2017; Ishimaru et al., 2019; Bakku et al., 2020). The investigation revealed that 314 MQTL genes (**Figure 3B**, **Supplementary Table 2**) were reported to be differentially expressed by Yamakawa and Hakata (2010). Similarly, 60, 56, 50, 48 and 26 genes were categorized as DEGs by Lin et al. (2017); Bakku et al. (2020), Ishimaru et al. (2019), Liu et al. (2010b) and Yamakawa et al. (2007), respectively (**Figure 3B**, **Supplementary Tables 3-7**). With an aim to discover a core set of chalk-associated genes we further shortlisted the DEGs on the basis of their presence in at least 2 expression datasets leading to the identification of 49 candidate genes (**Supplementary Table 8**; **Figure 3C**). Gene ontology (GO) analysis revealed that out of 49 candidates, 7 genes were involved in the carbohydrate metabolism. Besides these 9 genes belonged to the stress response pathway, 5 genes each belonged to the peptide metabolism pathway and seed storage proteins, 2 genes each were members of alpha amylase inhibitors, serine carboxypeptidases and carboxylic acid metabolic process. One member each was annotated as amino acid transporter and monosaccharide transporter, while 15 genes possessed non-significant annotations (**Figure 3D**).

### 4.4 Cis-regulatory elements in the promoter of candidate genes

**Figure 4:**
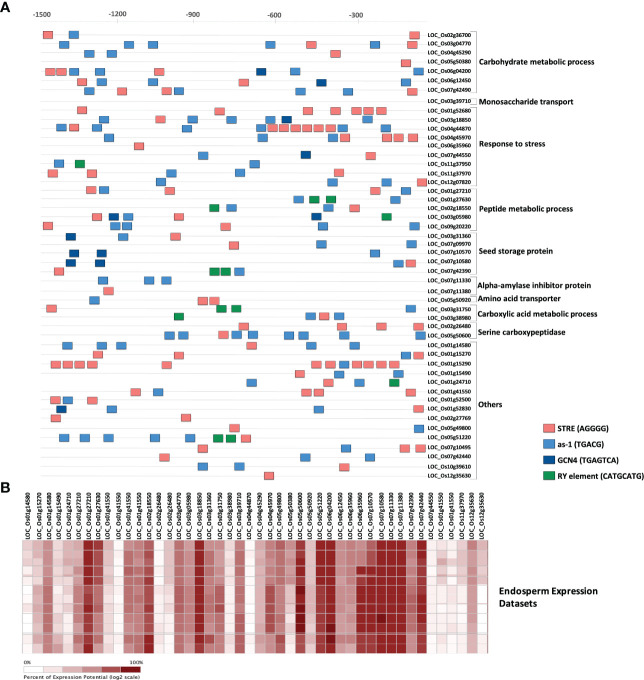
Spatio-temporal expression of candidate genes. **(A)** In silico identification of regulatory elements in the putative promoter region of the identified fundamental set and known chalk-responsive genes. Coloured boxes indicate as-1 element (blue), RY element (green), STRE (salmon) and GCN4 motif (dark blue) in 1500 bp upstream promoter regions. Scale is presented above the figure. **(B)** Heatmap highlighting the percent expression pattern of candidate genes in the endosperm, figure created using Genevestigator.

Although synthesis of starch is initiated in leaves where it serves for growth and development, seed endosperm being the sink is at the center of starch metabolism in rice (MacNeill et al., 2017). Consequently, many genes are co-expressed in the endosperm for regulating the optimal quality and quantity of starch granules. We, therefore, investigated whether the 49 candidate genes had common cis-elements in their promoters (**Figure 4A**) that might regulate their spatial and temporal expression. The 1.5 kb putative promoter region of all these genes were enriched with important cis-regulatory elements (CREs) elements, such as stress-responsive element (STRE; AGGGG), activation sequence-1 like promoter element (AS-1; TGACG), purine (A or G) and pyrimidine (C or T) element (RY; TGAGTCA) and GCN4 (CATGCATG). The AGGGG motif termed as stress-responsive element (STRE), initially identified in yeast, is responsive to several stresses (Martinez-Pastor et al., 1996). The AS-1 cis-element is present in genes regulated by auxin, MeJa and salicylic acid (Miao and Lam, 1995; Abdullah-Zawawi et al., 2021; Kabir et al., 2021). This element has also been reported in the promoters of several Glutathione-S-transferase genes (Hu et al., 2011) and abiotic stress response genes (Garretón et al., 2002). All the 49 investigated genes had the AS-1 and STRE motifs, suggesting that they might play a direct or indirect role in starch metabolism during unfavorable conditions. The GCN4 motif is crucial for seed-specific expression (Wu et al., 1998), while the RY element coordinates with other CREs and also plays a key role in seed-specific gene regulation (Ezcurra et al., 1999). A total of 17 genes either had GCN4 or RY motifs in their promoters indicating that these genes are possibly co-expressed in rice seeds. Further, with the help of Genevestigator expression datasets we observed that 41 out of 49 genes were differentially expressed in the rice endosperms (**Figure 4B**), indicating their role in the formation of rice grain chalk.

### 4.5 Haplotype analysis of 49 candidate genes reveals genetic diversity across the 3K rice genome panel

**Figure 5:**
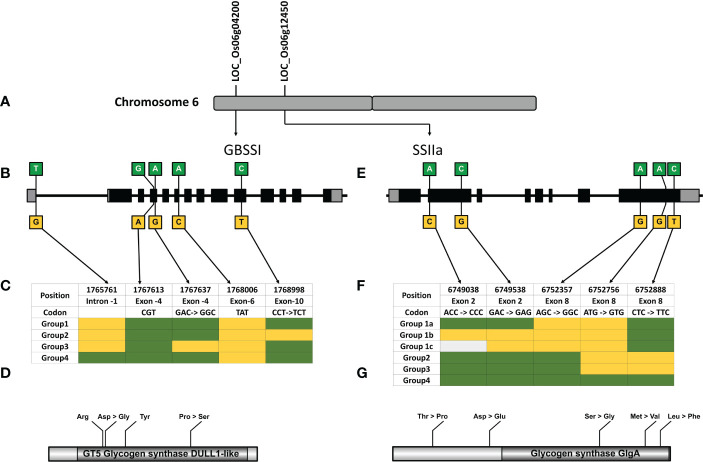
Haplotype analysis of Granule Bound Starch Synthase I (GBSSI) and Starch Synthase IIa(SSIIa). **(A)** The genes coding for GBSSI and SSIIa are located on the short arm of chromosome 6. **(B)** Gene diagram showing the non-synonymous variations of GBSSI, comprising 13 exons (grey boxes-UTR, Black boxes- CDS). **(C)** Haplotype table showing the SNP positions within GBSSI, four haplotypes are constructed based on the sequence variations in 25 rice genotypes (green indicates the reference allele Nipponbare, yellow indicates the SNPs and light grey shows missing data). **(D)** Depiction of GBSSI protein and amino acid changes in the GT-5 Glycogen Synthase DULL like domain predicted using NCBI CDD **(E)** Gene diagram showing the non-synonymous variations of SSIIagene comprised of 8 exons. **(F)** Haplotype table showing the SNP positions within SSIIa, four haplotypes, color codes same as **(B)**. **(G)** Depiction of SSIIa protein and amino acid changes in the Glycogen Synthase Glga domain predicted using NCBI CDD.

The 3K rice genome (RG) project is a giga dataset of publicly available genome sequences which can be used to mine genotypic variations in a population and link them to the phenotypic variability for exploring genetic diversity of different agronomic traits in rice (Li et al., 2014). We, therefore, performed a haplotype analysis of the 49 candidate genes to discover the non-synonymous variations in their coding sequences (CDS) across the 3K RG panel (**Supplementary Table 9**). Interestingly, 39 of the 49 analyzed candidate genes possessed non-synonymous SNPs ranging from 2 to 8, while the remaining 10 genes did not show any non-synonymous variations. Five of the seven carbohydrate metabolism-related genes contained non-synonymous SNPs in their coding sequences. We discovered 6 haplotypes and 1 allelic variation in sucrose transporter 5 (*SUT5*) and vacuolar invertase (*VIN2*), respectively, which were not reported previously and never before associated with the grain chalkiness and its related attributes. However, haplotypes in *GBSSI* and *SSIIa* have previously been implicated in starch metabolism and therefore we decided to explore the allelic variability in their CDS across the 3K panel and the subpanel of 60 accessions (**Figure 5**).

**Figure 6:**
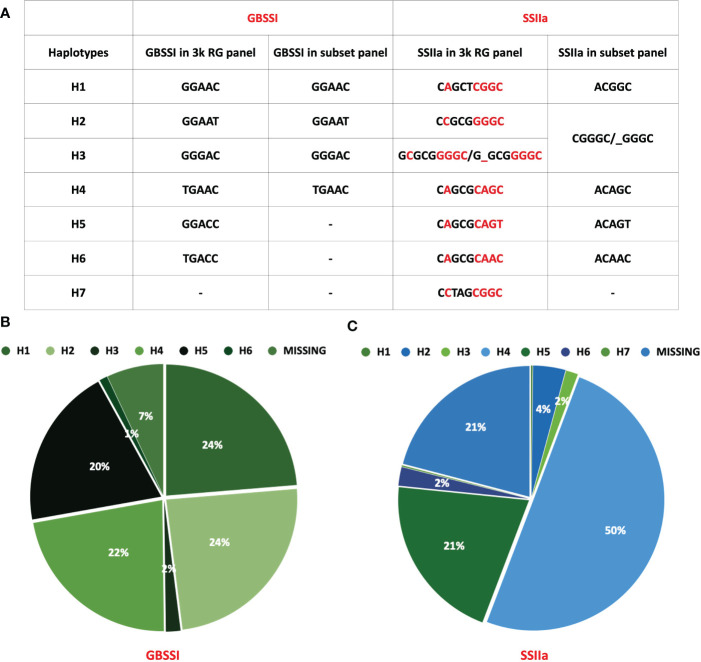
Distribution of haplotypes of GBSSI and SSIIa in rice 3k and its subset panel. **(A)** Table depicting the number of haplotypes in the 3k panel *Vs.* the subset panel for both genes, **(B)** Pie chart showing the % distribution of GBSSI haplotype in the 3k RG panel, **(C)** Pie chart highlighting the % distribution of haplotypes in SSIIa across 3k RG panel.

Waxy (Wx) gene, which encodes the enzyme *GBSSI*, converts ADP-glucose to amylose in the endosperm of cereals (Sano, 1984; Webb, 1991). Allelic variations of Wx gene in rice; Wx^lv^, Wx^a^, Wx^in^, Wx^b^, Wx^la/mw^, Wx^mq^, Wx^op/hp^ and wx (**Supplementary Table 10**), linked to varied amylose content (AC values), are distinguished by six polymorphic sites namely Int1-1, Ex2-112, Ex4-53, Ex4-77, Ex6-62, and Ex10-115 (**Figure 5B**) (Cai et al., 1998; Isshiki et al., 1998; Mikami et al., 1999; Sato et al., 2002; Wanchana et al., 2003; Mikami et al., 2008; Mikami et al., 2008; Liu et al., 2009; Yang et al., 2013; Zhang et al., 2019). *GBSSI* has 1 splice site SNP and 5 non-synonymous SNPs that give rise to 8 haplotype combinations. We were able to detect 6 of the 8 haplotype combinations in the entire 3K panel. Two hundred and one accessions (7%) had missing entries in the 3K panel either due to lower sequencing depth or sequencing errors and were therefore excluded from the analysis. Out of the remaining 2826 accessions, haplotype GGAAT (H2) had the highest frequency and was present in 738 accessions, whereas other haplotypes GGAAC (H1), TGAAC (H4), GGACC (H5), GGGAC (H3) and TGACC (H6) were observed in 23.7%, 22.4%, 19.8%, 1.8% and 1% of accessions, respectively (**Figure 6B**). A ‘G’ to ‘A’ change at Ex4-53 (Exon 4 th, 53 rd nucleotide) results in low amylose content and give rise to milky endosperms known as “milky queen” phenotype in seeds (Sato et al., 2002). Interestingly, the entire 3K panel had ‘G’ at Ex4-53 (**Figure 5C**) indicating that the “milky queen” phenotype was selected during varietal development. Owing to the presence of G in all the accessions, we were unable to find Wx^mq^ haplotype in the panel. Further, four haplotypes (GGAAC, GGAAT, GGGAC and TGAAC) were represented in the subset panel consisting of 60 accessions.

Soluble starch synthase IIa gene (*SSIIa*) elongates glucan chains from ADP-glucose units to form amylopectin in conjunction with branching and debranching enzymes (Denyer et al., 1995; Denyer et al., 1996; Tetlow and Emes, 2014). We discovered that *SSIIa* had 7 haplotypes arising from a combination of 9 non-synonymous SNPs (H1-CAGCTCGGC, H2-CCGCGGGGC, H3-GCGCGGGGC, H4-CAGCGCAGC, H5-CAGCGCAGT, H6-CAGCGCAAC, H7-CCTAGCGGC), in the entire 3K RG panel (**Figure 6A**). While 625 accessions had missing data, in the remaining 2402 accessions H4 was most predominant (50% of accessions). Other haplotypes in the descending order of their frequency (**Figure 6C**) were H7 (0.16%), H1 (0.2%), H3 (1.5%), H6 (2.3%), H2 (4%) and H5 (20.8%). However, we could only detect 5 SNPs giving rise to 6 haplotypes in the subset panel of 60 accessions (Figurse 6 e, f). The missing 4 SNPs C, G/T, C/A and G/T resulted in 3 combinations “CGCT” (H1), “CGCG” (H2-H6) and “CTAG” (H7). While the combination of missing SNP “CGCT” was present in 21% of accessions, “CTAG” was present in only 2% of accessions. Out of the 6 haplotype combinations in the gene encoding for SSII-a enzyme, 4 haplotype combinations arising from 3 SNPs (H1-GGC, H2-AGC, H3-AGT and H4-AAC), are associated with chain length of amylopectin, activity of starch synthase, gelatinization temperature and alkali spread score (Umemoto et al., 2004; Umemoto and Aoki, 2005; Waters et al., 2006). All the 3 SNPs resulting in 4 haplotypes are present in the catalytic domain of the enzyme (**Figure 5G**) and therefore are important for enzyme activity, which was more for the haplotypes H1 and H2 and less for H3 and H4 (Umemoto et al., 2004; Umemoto and Aoki, 2005).

### 4.6 Identification of superior haplotypes for granule bound starch synthase I and starch synthase IIa by haplo-pheno analysis

**Figure 7:**
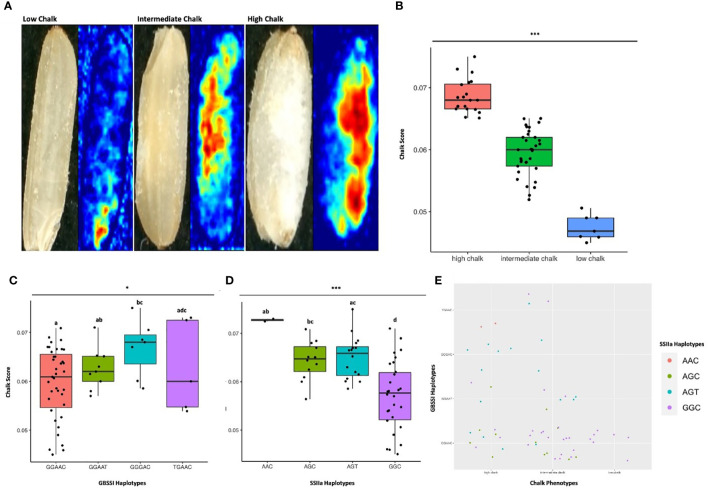
Haplo-pheno analysis of Granule Bound Starch Synthase I and Starch Synthase II a. **(A)** Rice seed scans and their corresponding heat maps highlighting the chalky area (red) in less chalk, intermediate chalk and high chalk seed types. **(B)** Box plot depicting the chalky score of 60 rice genotypes (grown in two crop seasons) categorized as high, intermediate and low chalk. **(C)** Box plot of the chalk score data categorized according to the GBSS I haplotypes. **(D)** Box plot of the chalk score data categorized according to the SSIIa haplotypes. **(E)** Comparison of the haplotypic combination of GBSS I and SSIIa. One-way ANOVA was conducted for determining the statistical significance of haplotype means. Chalk score data designated with the same alphabet are not significantly different at p ≤ 0.1 (.) or p ≤ 0.05 (*) or p ≤ 0.001 (***) as per Tukey *Post hoc* test for Honest Significance Difference (HSD) analysis.

Since haplotypes in *GBSSI* and *SSIIa* are important for their activity and likely to affect starch quality, we investigated their haplotype behavior with respect to the grain chalkiness in the subset panel of 60 accessions. These accessions were subjected to high temperature stress under natural field conditions during the grain filling stages over two Rabi seasons in Raipur, India. The amount of chalk (expressed as “chalk score”) was determined by scanning the polished rice grains and processing the images through a Gradient-weighted Class Activation Mapping (Grad-CAM) tool (Wang et al., 2022). The chalk score ranged from 0.046 to 0.075 and the accessions were distributed in low chalk (CS ≤ 0.05), intermediate chalk (0.05 > CS ≤ 0.065) and high chalk (CS > 0.065) categories (**Supplementary Table 11**, **Figure 7A**). Seven accessions were in the low chalk category, thirty-three in the intermediate and twenty were in the high chalk category. Inferential statistics ascertained that the means of all three chalk categories were significantly different (**Figure 7B**). Analysis of variance (ANOVA) determined that the variability of chalk score with respect to all the 4 haplotypes of SSIIa and GBSSI were significantly different and had p-values of ≤ 0.05 and ≤ 0.1, respectively. However, after Tukey’s *post hoc* analyses, differences in the mean CS values for only *GBSSI* haplotypes GGAAC and GGGAC were significant (**Figure 7C**). Out of the 4 possible haplotypes in the catalytic domain of *SSIIa*, significant differences in the mean CS values were seen between GGC and AGT, GGC and AGC and GGC and AAC (**Figure 7D**). To find whether and which combination of haplotypes for *GBSSI* and *SSIIa* associate with low grain chalkiness under high temperature stress, we performed a correlation analysis of different haplotype combinations with the chalkiness phenotypes. Interestingly, all the seven accessions with lowest mean CS, had the haplotypic combination of GGAAC (for *GBSSI*) and GGC (for *SSIIa*). Fifteen accessions having an intermediate CS, also had a similar haplotype combination. The other 18 accessions with intermediate CS had the following haplotype combinations for *GBSSI*/*SSIIa*; GGAAC/AGC, GGAAT/GGC, GGAAT/AGC, GGAAT/AGT, GGGAC/AGT, GGAAC/GGC, TGAAC/GGC and TGAAC/AGT. Five high CS accessions had the haplotype combinations (*GBSSI*/*SSIIa*) of GGGAC/AGT, while four had GGAAC/AGC, three each had GGAAC/AGT and GGAAC/GGC, two had TGAAC/AAC and 1 each had GGAAT/AGC, GGAAT/AGT and GGAAT/GGC.

### 4.7 Scanning electron microscopy validated the correlation between chalk score and haplotypic combinations

**Figure 8:**
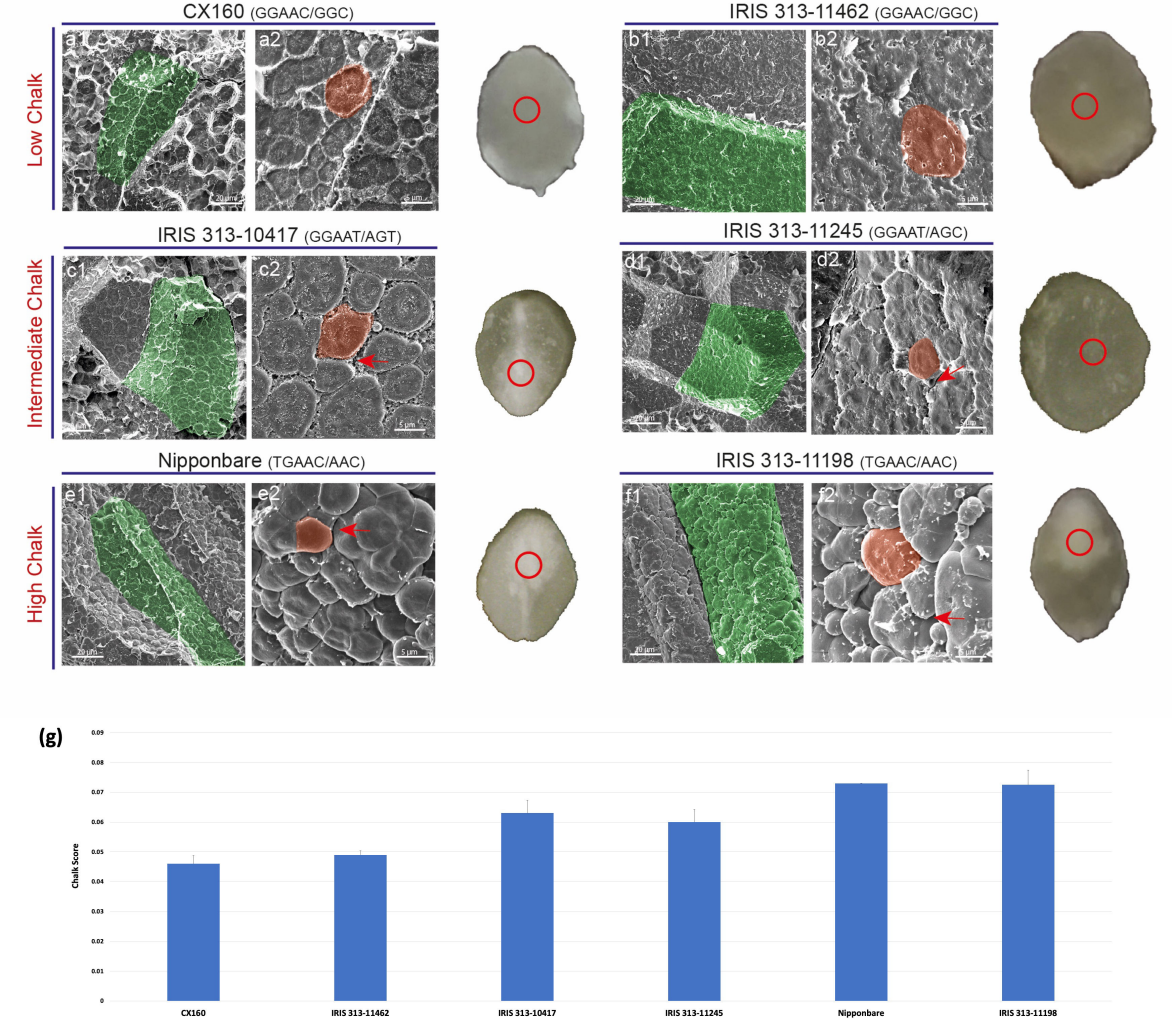
Scanning electron micrographs of transversely fractured mature rice seeds. (A1-B2) Endosperm cell (green) of low chalk genotypes is tightly packed with starch grains (orange). (C1-D1) Micrographs of intermediate chalk genotypes with slightly bulged starch granules with visible spaces between individual amyloplasts. (E1-F2) Images of transversely cut chalky endosperm region filled with spherical starch grains instead of polygonal as in a1 and b1. Air spaces (shown by red arrows) can be seen between the grains in e1 and f1. The red circle on a transverse cut seed shows the region selected for analysis.

Six accessions, two from each category of low, intermediate and high chalk score were selected in order to find correlation between haplotypes and packaging of starch grains in chalky and non-chalky seeds. The seeds of the accessions CX160 and IRIS 313-11462 having low chalk score with GGAAC/GGC haplotypic combination showed dense packaging of starch granules under scanning electron microscope. The amyloplast and the starch grains in them were very tightly packed and no space was visible between them. This probably contributed to the translucent appearance of the grain (**Figures 8A1, B1**). Whereas, the accessions IRIS 313-10417 and IRIS 313-11245, with GGAAT/AGC, GGAAT/AGT haplotypic combination grouped under the intermediate chalky category, exhibited slightly bulged starch granules with visible spaces between individual amyloplasts (shown with red arrow, **Figures 8C1, D1**). In contrast, the high chalk genotypes Nipponbare and IRIS 313-11198 with TGAAC/AAC and TGAAC/AAC haplotypic combinations showed visibly round starch granules (indicative of loose packaging) instead of polyhedral and with noticeable air spaces between them. Presence of air spaces and loose packaging scatters light leading to opaque or chalky appearance as shown in **Figures 8E1, F1**. A magnified view of the starch granules is shown in the lower panel (**Figures 8A2-F2**). The SEM data validates a clear association between haplotypic combinations and phenotypic data relating to degree of seed chalkiness.

## 5 Discussion

Mapping of quantitative trait linked genomic regions in segregating populations from diverse genetic backgrounds has led to the identification of many grain chalk associated QTLs. Majority of these studies employed markers providing low genetic resolution and therefore the identified QTLs span a large CI. Furthermore, many QTLs from independent studies map to similar genomic regions. It is, therefore, important that common and consistent regions of overlapping QTLs are identified for their effective utilization in trait improvement programs. A meta-QTL analysis aptly serves to achieve this goal (Goffinet and Gerber, 2000) and has been successfully applied to several crop plants, including rice. In this study, we performed a meta-analysis of rice grain chalk QTLs to precisely identify the most significant QTL regions and their underlying candidate genes. We were able to restrict the most important genetic information of 403 QTLs within 64 meta-QTLs. To comprehensively identify chalk-associated candidate genes, expression of the genes underlying these meta-QTLs was assessed in the published grain chalk-associated expression datasets, leading to the identification of a core set of 49 genes. We then performed a promoter analysis to identify the common cis-regulating elements, followed by a haplotype analysis of the 49 candidate genes across the 3K RG panel which revealed natural variations in these genes. Owing to the direct connection of genes involved in sugar transport and metabolism, we correlated haplotypes in 6 sugar metabolism genes with the grain chalkiness in a subset of 60 accessions. Our analysis revealed haplotype combinations in *GBSSI* and *SSIIa* that are associated with presence of low chalk.

### 5.1 Meta-analysis complemented with gene expression underpins key genes involved in grain starch metabolism

**Figure 9:**
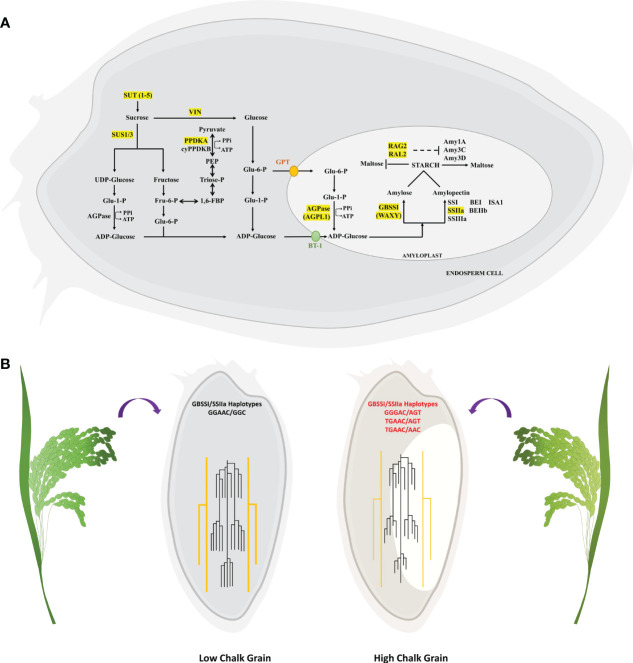
Depiction of identified candidate genes in the starch metabolism pathway and their haplotypic combinations affecting grain chalkiness. **(A)** Starch metabolism pathway in seeds begins with the sucrose transporters (SUTs) that translocate sucrose into the seeds, followed by its breakdown into glucose and fructose by the enzymatic action of invertases (VIN) and sucrose synthases (SUS). Finally glucose-1-phosphate (Glu-6-P) is converted to ADP-glucose by ADP-glucose pyrophosphorylase(AGPase), which is converted to starch by the combined action of Granule Bound Starch Synthase I (GBSSI) which forms amylose and Starch Synthase IIa (SSIIa) which forms linear glucan chains leading to formation of amylopectin. During HT amylases (Amy) breakdown starch to malto-oligosaccharides. RAG2/RAL2 from alpha-amylase inhibitor family are thought to suppress the amylase activity. **(B)** Grains with low chalk have high amylose and longer chain length while high chalk grains have low amylose and short chain amylopectin. Low grain chalk seeds have superior haplotypic combination of GBSSI and SSIIa when compared with high chalk seeds.

Conversion to starch from UDP/ADP-Glucose is a multistep process that begins with the transport of sucrose, its conversion to glucose followed by synthesis of amylose and amylopectin (**Figure 9A**). Genes and proteins involved in each of these steps are themselves regulated at transcriptional, post-transcriptional and post-translational levels. Majority of the QTL mapping studies for grain chalk have identified genes directly involved in starch synthesis, while other studies have led to the discovery of genes which code for regulators. In the MQTL analysis we identified 7 genes that code for proteins belonging to the starch metabolism pathway, owing to their discovery in overlapping QTLs derived from different mapping studies. This further proves that regulation of sugar metabolism governs the core process of chalk formation.

Sucrose transporters (*SUTs*) mediate the proton symport coupled transport of sucrose across the plasma membrane through the phloem to developing grains (Aoki et al., 2003). The *OsSUT* gene family comprises five members *OsSUT* 1, 2, 3, 4 and 5 that demonstrate overlapping expression patterns during grain filling and therefore might exhibit partial functional redundancy (Aoki et al., 2003). *OsSUT*5, one of the candidate sugar metabolism gene identified in the current study lies in MQTL 2.4, which is contributed by QTLs qPGWC2 (Chen et al., 2011), qAEC2 and qDEC2 (Wan et al., 2005). Structure-based modeling experiments and electrophysiology assays in Xenopus oocytes revealed that five of the six charged amino acids (D177, R188, D331, R335 and E336) are important for sucrose transport by *OsSUT1* (Sun et al., 2012). All the five functionally important amino acids were conserved in *OsSUT5* (unpublished data) across the 3K panel. This signifies the importance of functional redundancy in sucrose transporters for mobilization of sugars to sink to ensure normal grain filling. Further, haplotype analysis revealed 6 non-synonymous haplotypes of *SUT5* gene in the entire 3K panel (**Supplementary Table 9**). However, none of the haplotypes were in the wobble base of the codons for the five functionally important amino acids. None of the haplotypes grouped with the chalk score data in the 60 accessions which implies that their relevance will need further experimentation. Nonetheless, the formation of grain chalk is regulated by a aleurone-specific TF, *OsNF-YB1*, which binds to the promoters of *OsSUT* genes and transcriptionally activates their expression (Bai et al., 2016). In this respect, it would therefore be important to assess the polymorphism at “CCAAT” boxes in the promoters of *OsSUT* genes in future. Sucrose synthase (SUS) enzyme is encoded by 7 conserved *SUS* genes with compensatory functions (Hirose et al., 2008; Cho et al., 2011) and catalyzes the conversion of sucrose and uridine diphosphate (UDP) into fructose and UDP-glucose (Huang et al., 2016). *OsSUS3*, a candidate gene in this study present in MQTL 7.4 [contributing QTLs qWBA, qWCA, qWBR and qET(Peng et al., 2014a; Yun et al., 2016; Mei et al., 2013)] was predominantly expressed in the endosperm and the aleurone layers at the onset of starch synthesis (Huang et al., 1996). Our data revealed that the *SUS3* gene had 1 allelic variation in the coding region of the 3K panel (**Supplementary Table 9**), which is in contrast to previous reports where presence of 2 (6 SNPs) and 11 haplotypes in the coding sequence have been reported (Lestari et al., 2011; Takehara et al., 2018). Additionally, haplotypes were also identified in the non-coding region of the *SUS3* gene which included the promoter (Lestari et al., 2011; Takehara et al., 2018). It was, however, surprising that none of the previously identified haplotypes in the *SUS3* CDS were seen in the 3K panel. Only 3 of the varieties (IR64, Milyang 23 and Lemont) harboring the reported haplotypes were part of the 3K sequenced panel. We believe that these haplotypes were not detected in the variant analysis of the 3K panel because the SNP dataset was a filtered dataset [cutoff values of minor allele frequency (MAF) > 1% and missing < 20%; Mansueto et al., 2016]. Nonetheless the *SUS3* alleles in the promoter and coding region from Habataki, an indica variety were important for low grain chalk under high temperature stress as introgression of Habataki SUS3 alleles in Koshihikari (Apq1-NIL), a japonica variety, led to increased percentage of perfect grains (Takehara et al., 2018).

Sucrose flux in the plant metabolic pathways is regulated by isoforms of invertases (Sturm, 1999) which catalyze the irreversible cleavage of sucrose into glucose and fructose. Rice has eight neutral invertases (*NINs*, cytosolic), nine cell wall invertases (*CWINs*) and two vacuolar invertases (*VINs*). Our study identified *OsINV2* as one of the candidate genes in MQTL 4.4 [contributed by QTLs qDPGWC, qPGWC, qWCR4.1, qWCR4.2, qch (Peng et al., 2014a; Wang et al., 2016; Yun et al., 2016; Edwards et al., 2017)]. *OsVIN2* and its homologue *OsVIN3* regulate the sink size by controlling hexose to sucrose ratio and drive cell expansion by turgor generated due to influx of osmotic solutes (Koch, 2004; Sergeeva et al., 2006; Morey et al., 2019). Consequently, *vin2* and *vin3* mutations lead to decreased seed size and shorter and lighter panicles (Morey et al., 2018; Lee et al., 2019; Morey et al., 2019; Xu et al., 2019; Deng et al., 2020). Other than being present as a candidate gene in the above-mentioned grain chalk-associated QTLs, *OsVIN2* has not been directly implicated in the grain chalkiness. However, the *OsCWIN* member *OsCIN2* mapped to the grain incomplete filling 1 (*OsGIF1*) locus was associated with grain filling and chalkiness in rice (Wang et al., 2008; Wang et al., 2018). Expression of GIF1 is unaffected, whereas both *OsVIN2* and *VIN3* are down regulated by high temperature and therefore it is plausible that the reduced expression of *VINs* contribute to reduced starch synthesis leading to increased grain chalkiness.

Glucose and fructose are also generated from phosphoenolpyruvate (PEP) which is synthesized by the action of pyruvate orthophosphate dikinase (*PPDK*) enzyme. Rice, a C3 plant has 3 *PPDK* genes, *OsPPDKA* encoding a cytosolic isoform (*OsPPDKA*); *OsPPDKB*, expressing a cytosolic *PPDKB* (cy*OsPPDKB*); and the chloroplastic *PPDK* (ch*OsPPDKB*) (Imaizumi et al., 1997; Moons et al., 1998). Our study identified *OsPPDKA* as one of the candidate genes from MQTL 3.5 [contributing QTLs qPGWC3.1, qPGWC3.2, qWB, qWBA, qWBK, qET and qCA (Kobayashi et al., 2007; Mei et al., 2013; Kobayashi et al., 2013; Peng et al., 2014a; Wang et al., 2016)]. It had only 2 non-synonymous haplotypes in the 3K panel, which could not be correlated with the chalk phenotype. Mutation in cy*PPDKB* gene resulted in chalky grains and a floury endosperm (Kang et al., 2005). As compared to *PPDKA*, *PPDKB* has a higher expression during the milky stage in rice kernels (Kang et al., 2005). On the other hand, *PPDKB* is down-regulated, while *PPDKA* is induced in response to heat stress during grain filling in Nipponbare (Yamakawa et al., 2007). We believe that *PPDKB* and *PPDKA* have compensatory roles and their stoichiometric levels determine quantity and quality of starch under high temperature. However, this hypothesis needs further validation by comparing the relative expression of *PPDKA* and *PPDKB* in rice accessions having contrasting grain chalk phenotypes.

One of the key regulatory steps in starch biosynthesis is conversion of glucose-1-phosphate to ADP-glucose catalyzed by the heterotetrameric enzyme complex (Okita, 1992; Tetlow et al., 2004; Lee et al., 2007). The heterotetramer has 2 small (*OsAGPS1* and *AGPS2a/b*) and 2 large subunits (*AGPL1-4*). *AGPL1*, was identified as a candidate gene in MQTL 5.5 [contributed by QTLs qPGWC5.1 and qPGWC5.2 (Chen et al., 2016; Wang et al., 2016)]. Interestingly, we did not find any non-synonymous variations in *AGPL1* throughout the 3K rice genome panel. This is in agreement with Lu and Park (2012) who also could not find any variation in the *AGPL1* gene in 104 rice accessions. ADP-glucose pyrophosphorylase genes, *AGPL2* and *AGPS2b*, exhibit polymorphisms that do not alter their protein (Kharabian-Masouleh et al., 2011; Wei et al., 2017) and conservation of protein sequence is likely a general feature of all the ADP-glucose pyrophosphorylase enzyme subunits. *AGPL1* is localized in amyloplasts and is expressed in the early stages of grain filling, whereas *AGPL2* is localized to cytosol and is expressed in the middle to late stages of grain filling (Lee et al., 2007) and it is likely that their spatio-temporal expression determines the quantity and quality of starch.

Plants exposed to high temperatures require energy to maintain cellular homoeostasis (Sadok and Jagadish, 2020) for which it breaks down reserve starch by increasing the expression and activity of α-amylases in the ripening grains. The timing of HT stress is crucial because if it happens during the grain filling stages, increased expression and activity of α-amylases affects the formation of starch granules and at the same time increases the hydrolysis of the already deposited starch, ultimately producing chalky grains (Zakaria et al., 2002; Iwasawa et al., 2009). RNAi-mediated suppression of α-amylases reduces grain chalk formation under high temperature, conversely their overexpression enhances chalk even under ambient temperatures (Asatsuma et al., 2006; Hakata et al., 2012; Nakata et al., 2017). *RAG2*, a candidate gene from MQTL7.1 [contributing QTLs qDPGWC7.1, qDPGWC7.2, qPGWC7.1, qPGWC7.2, qPGWC7.3 (Wang et al., 2016)] is a 16-kDa alpha-amylase/trypsin inhibitor in rice. It is expressed in the developing seeds and is involved in both the regulation of grain yield and quality (Zhou et al., 2017). Rice grains of *RAG*2-RNAi lines display high chalk with loosely packed starch as compared to the wild type (Zhou et al., 2017). *RAL2*, also a candidate gene from MQTL 7.1, displays high homology with *RAG2* and may have a similar function. We hypothesize that both *RAG2* and *RAL2* proteins might inhibit α-amylase activity under high temperature but further experimental validation is needed. Our study revealed that *RAG2* and *RAL2* genes have 4 and 2 haplotypes respectively, in the 3K RG panel. We found 2 haplotypes each for *RAG2* and *RAL2* in the subset panel of 60 accessions, which did not correlate with the degree of chalkiness.

We also discovered β-amylase, another starch hydrolyzing enzyme of amylase family, as a candidate gene from MQTL 3.1 [contributing QTLs qWCR, qBW, qWB, qPGWC3.1 and qPGWC 3.2 (Chen et al., 2011; Wada et al., 2015; Wang et al., 2016; Yun et al., 2016)]. Haplotype analysis in the 3K RG panel revealed that beta-amylase is conserved and had no haplotypic variations. The expression of beta-amylase increases under high temperature (Yamakawa et al., 2007) and its activity in chalky grains is higher than in non-chalky grains (Nakamura et al., 2021). An endosperm-specific β-amylase gene from barley, *ESDBamy* is expressed in early endosperm and a shrunken endosperm mutant shows reduced starch deposition due to high levels of its expression (Jung et al., 2001). Absence of non-synonymous haplotypes indicate that differential steady state mRNA levels of β-amylase gene might be a contributing factor for variable amounts of starch in different accessions by inequitably affecting the inhibition of starch deposition. Both *RAG2* and *RAL2* are induced by high temperature and it is plausible that α- and β-amylases are targets of *RAG2* and *RAL2*, respectively. Further experiments involving expression analysis of these genes in multiple accessions with varying degrees of chalk, protein-protein interactions and enzymatic assays will be required to validate these possibilities and possibly provide additional strategies to regulate formation of grain chalk in rice.

Another major group of candidate genes were either seed storage proteins or those involved in nitrogen metabolism. We identified 5 genes each for seed storage proteins and nitrogen metabolism pathway in our meta-QTL analysis. Since both starch and proteins in grains are synthesized using sugar as the carbon source (Bao et al., 2015; Xin et al., 2019), an optimal coordination between the C and N metabolism during grain filling is crucial, but less worked upon. Reports suggest that the nitrogen metabolism pathway and synthesis of storage proteins in particular may lead to grain chalk formation in rice (Han et al., 2012; Xi et al., 2021). A significant negative correlation between prolamin content and chalkiness has also been discovered (Wang et al., 2021). It would be interesting to systematically investigate these promising candidate genes to define their roles in grain chalkiness.

### 5.2 Natural variations of GBSSI and SSIIa influence the quality of rice grains

Amylose and amylopectin synthesis in the rice endosperm are regulated GBSSI and SSIIa enzymes, respectively, genes for which are located on chromosome 6 (Wang et al., 1990, **Figure 5A**). *GBSSI*, a candidate gene identified in the current study from MQTL 6.1, was found in 14 mapped QTLs [qWCR, qCR, qWBA, qPCG1, qPCG2, qDC1, qDC2, qDC3, qACE, qPGWC1, qPGWC2, qCA, qWC and qWB (Peng et al., 2014a; Mei et al., 2013; Kobayashi et al., 2007; Zheng et al., 2012; Lu et al., 2013; Gao et al., 2016a)]. Similarly, MQTL 6.3 from which *SSIIa* gene was identified as a candidate, was derived from 7 overlapping QTLs [qDPGWC, qWCR, qCA, qET, qCA, qch, qWBR (Wang et al., 2016; Mei et al., 2013; Tan et al., 2000; Peng et al., 2014a; Yun et al., 2016)]. Such a high number of QTLs from many different studies signifies that these genomic regions and the underlying candidate genes immensely contribute to the grain chalk trait.

It is known that the diversity of grain amylose content is largely due to allelic variations at the Wx locus (Tian et al., 2009; Biselli et al., 2014; Zhang et al., 2019). To date, at least eight Wx alleles, Wx^lv^, Wx^a^, Wx^in^, Wx^b^, Wx^op/hp^, Wx^mq^, Wx^mp^, and wx, have been shown to be associated with the five possible amylose types observed in rice cultivars (Cai et al., 1998; Sato et al., 2002; Larkin and Park, 2003; Wanchana et al., 2003; Mikami et al., 2008; Liu et al., 2009; Yang et al., 2013; Zhang et al., 2019). In the present study we identified Wx alleles, Wx^lv^, Wx^a^, Wx^b^ and Wx^op/hp^ leading to the detection of 4 *GBSSI* haplotypes. Previously Wx^lv^ has been associated with high amylose content (**Supplementary Table 10**, Zhang et al., 2019), which could result in a low chalk accumulation phenotype. Wx^lv^ corresponded with 4 low, 4 intermediate and 3 high chalk phenotypes in our study, indicating that allelic combinations of other alleles with the Wx^lv^ allele possibly determines the degree of chalkiness. While Wx^a^ allele was primarily associated with 5 intermediate and 1 high chalk type rice, accessions having high chalk almost always had Wx^b^ and Wx^op/hp^ alleles.

Most of the indica rice cultivars possess active-type *SSIIa* (wild-type), whereas typical japonica rice cultivars possess mutant *SSIIa* harboring three amino acid substitutions in the active site, thus exhibiting only 10% of *SSIIa* activity relative to indica rice (Nakamura et al., 2005). Low SSIIa activity in japonica rice leads to a reduction in amylopectin branches with degree of polymerization (DP) of 13-24, and an increase in short amylopectin chains with DP ≤ 12 (Umemoto et al., 1999; Umemoto et al., 2002; Nakamura et al., 2005). The four haplotypic combinations in our study were derived from the three substitutions. The GGC haplotype was associated with low and intermediate chalk accessions, intermediate chalk accessions had AGC, while AGT and AAC were linked with high chalk accessions, indicating that a combination of substitutions in the active site of SSIIa enzyme is a critical determinant of chalk formation.

Since, elevated temperatures affect the accumulation of starch (Umemoto et al., 1995) by disturbing the proportion of amylose to amylopectin (Inouchi et al., 2000), therefore, understanding how various allelic combinations of closely located starch biosynthetic genes, *GBSSI* and *SSIIa*, affect starch properties and grain chalkiness is of utmost importance. Haplotypes GGGAC, TGAAC in *GBSSI* (Cai et al., 1998; Mikami et al., 1999; Larkin and Park, 2003) and AGT and AAC in *SSIIa* (Umemoto et al., 1999; Umemoto et al., 2002; Nakamura et al., 2005) have low amylose and low amylopectin. Our chalky score and scanning electron microscopy data revealed that the combinations GGGAC/AGT, TGAAC/AGT and TGAAC/AAC in accessions invariably led to high chalk accumulation (**Figure 9B**). Starch quality in rice grains is a trait that has evolved in response to strong selection of starch synthesis pathway genes during domestication (Whitt et al., 2002). Rice varieties with high amylose levels (20–30%) tend to form discrete, non-cohesive grains when cooked and are found in South and Southeast Asian rices classified as indica and tropical japonica variety groups, whereas varieties with low amylose, a characteristic of temperate japonica variety, are common in northeast Asia and form cohesive cooked grains (Morishima and Sano, 1992; Juliano and Villareal, 1993). Because *GBSSI* and *SSIIa* are closely located on chromosome 6, it is possible that these two genes may have undergone co-selection during domestication. Further, rice germplasm harboring different haplotypic combinations of these starch synthesizing genes have likely been selected on the basis of regional palatability. Nevertheless, the haplotype combinations associated with low grain chalk will be helpful in developing elite varieties with high head rice yield.

## 6 Conclusions and future directions

Meta-QTL analysis is a powerful tool for detecting the most precise and concurrent QTLs for any agronomic trait in crops. Identification of differentially expressed MQTL genes utilizing trait-specific transcriptome datasets, provides additional means for refining the process of shortlisting the significant candidate genes. Harnessing information of haplotypic diversity from sequenced accessions and associating it with the phenotype can provide information on novel allelic/haplotype combinations contributing to traits of economic importance. Our work combined all these approaches and unveiled haplotype combinations in two main starch synthesis genes, which can be genetically linked with varying degrees of grain chalkiness in rice. The favorable allelic combinations that promote biosynthesis of optimum quality starch and thereby contribute to reduced grain chalk, can be backcrossed with high-yielding elite varieties or CRISPR-Cas based prime editing to generate superior rice plants with low grain chalkiness and high HRY traits.

## Data availability statement

The original contributions presented in the study are included in the article/**Supplementary Material**. Further inquiries can be directed to the corresponding author.

## Author contributions

MA and AK planned and designed the research. AK, DS, and KK performed the meta-analysis. AK and DS mined the candidate genes and performed the in-silico promoter analysis. AK and S analyzed expression data. AK, DS, CW, DC, AP, GC, and MS did the phenotyping. AK, MI, and PK performed the haplotype analysis. AK and PS performed the haplo-pheno analysis. SK and VV prepared the SEM images. AK, MA, SK-A, AG, and SJ wrote the manuscript. All authors contributed to the article and approved the submitted version.
