# Erratum: Meta-QTL and haplo-pheno analysis reveal superior haplotype combinations associated with low grain chalkiness under high temperature in rice

**DOI:** 10.3389/fpls.2023.1254738

**Published:** 2023-07-17

**Authors:** Frontiers Production Office

**Affiliations:** Frontiers Media SA, Lausanne, Switzerland

**Keywords:** starch metabolism, grain chalkiness, meta-QTL analysis, haplotype, haplo-pheno analysis, granule bound starch synthase I, starch synthase IIa

Due to a production error, **Figure 1** was a repeat of **Figure 7** in the published article.

The corrected Figures appear below.

**Figure 1:**
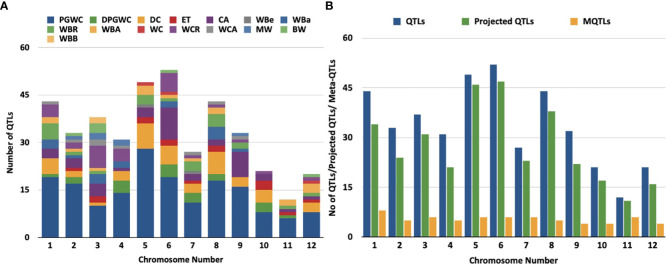
Distribution of QTLs and meta-QTLs associated with rice grain chalk on different chmmosomes of rice, **(A)** Trait-wise distribution of initial QTLs used for the meta-QTL analysis, (PGWC, Percentage Grain with Chalkiness; DPGWC, Degree of Percentage Grain with Chalkiness; DC, Degree of Endosperm Chalkiness; ET, Endosperm Transparency; CA, Chalkiness Area; WBe, White Belly; WBa, White Back; WBR,; WBA, White Back Area; WC, White core; WCR, White core rate; WCA, Whlte core area; MW,Mi1ky white; BW, Basal White; WBB, White Back and Basal). **(B)** The distribution of QTLs, projected QTLs and meta-QTLs on twelve rice chromosomes.

**Figure 7:**
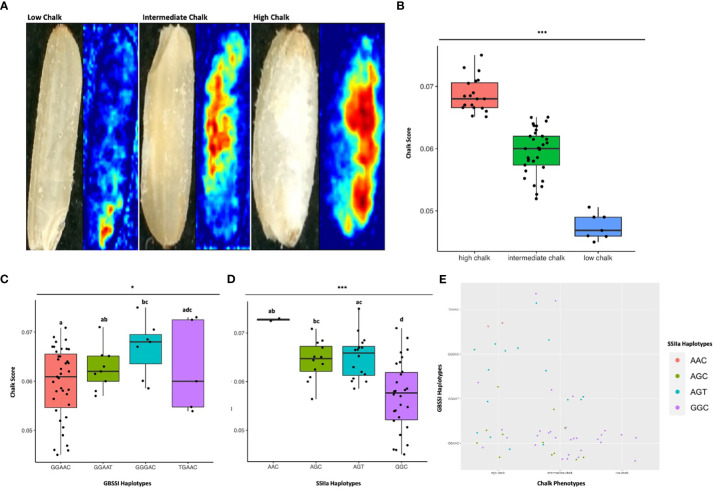
Haplo-pheno analysis of Granule Bound Starch Synthase I and Starch Synthase Il a, **(A)** Rice seed scans and their corresponding heat maps highlighting the chalky area (red) in less chalk, intermediate chalk and high chalk seed types. **(B)** Box plot depicting the chalky score of 60 rice genotypes (grown in two crop seasons) categorized as high, intermediate and low chalk **(C)** Box plot of the chalk score data categorized according to the GBSS I haplotypes. **(D)** Box plot of the chalk score data categorized according to the SSIIa haplotypes. **(E)** Comparison of the haplotypic combination of GBSS I and SSIIa. One-way ANOVA was conducted for determining the statistical significance of haplotype means. Chalk score data designated with the same alphabet are not significantly different at p ≤ 0.1(.) or p ≤ 0.05 (*) or p ≤ 0.001 (***) as per Turkey *Post hoc* test for Honest Significance Difference (HSD) analysis.

The publisher apologizes for this mistake.

The original version of this article has been updated.

