# Supplementary figures and images for: Meta-QTL and haplo-pheno analysis reveal superior haplotype combinations associated with low grain chalkiness under high temperature in rice
Source: Front Plant Sci. 2023 Mar 8;14:1133115. doi: 10.3389/fpls.2023.1133115 (PMC10031497; doi:10.3389/fpls.2023.1133115)

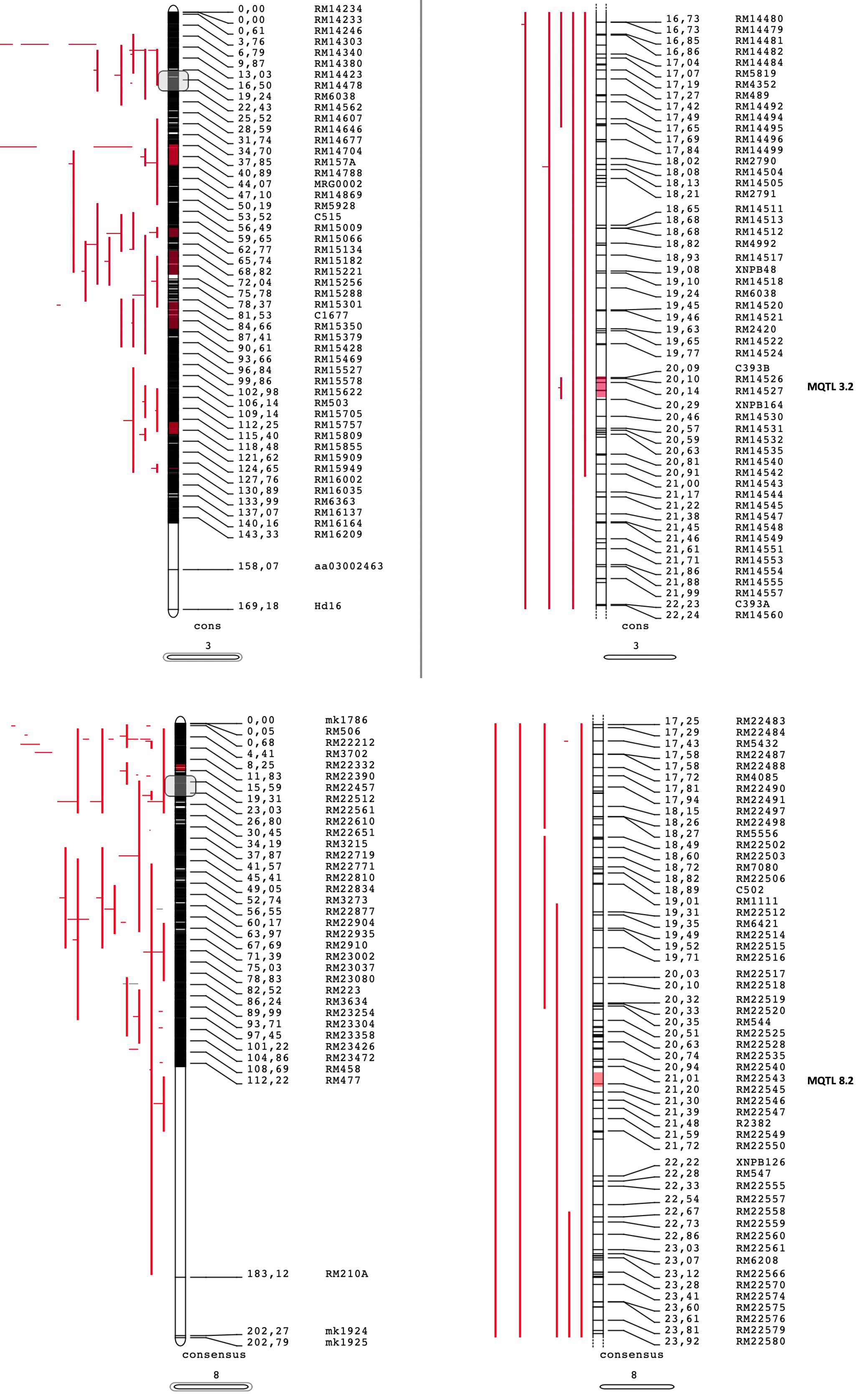

Supplement: Supplementary file 1 [file Image_1.jpeg]

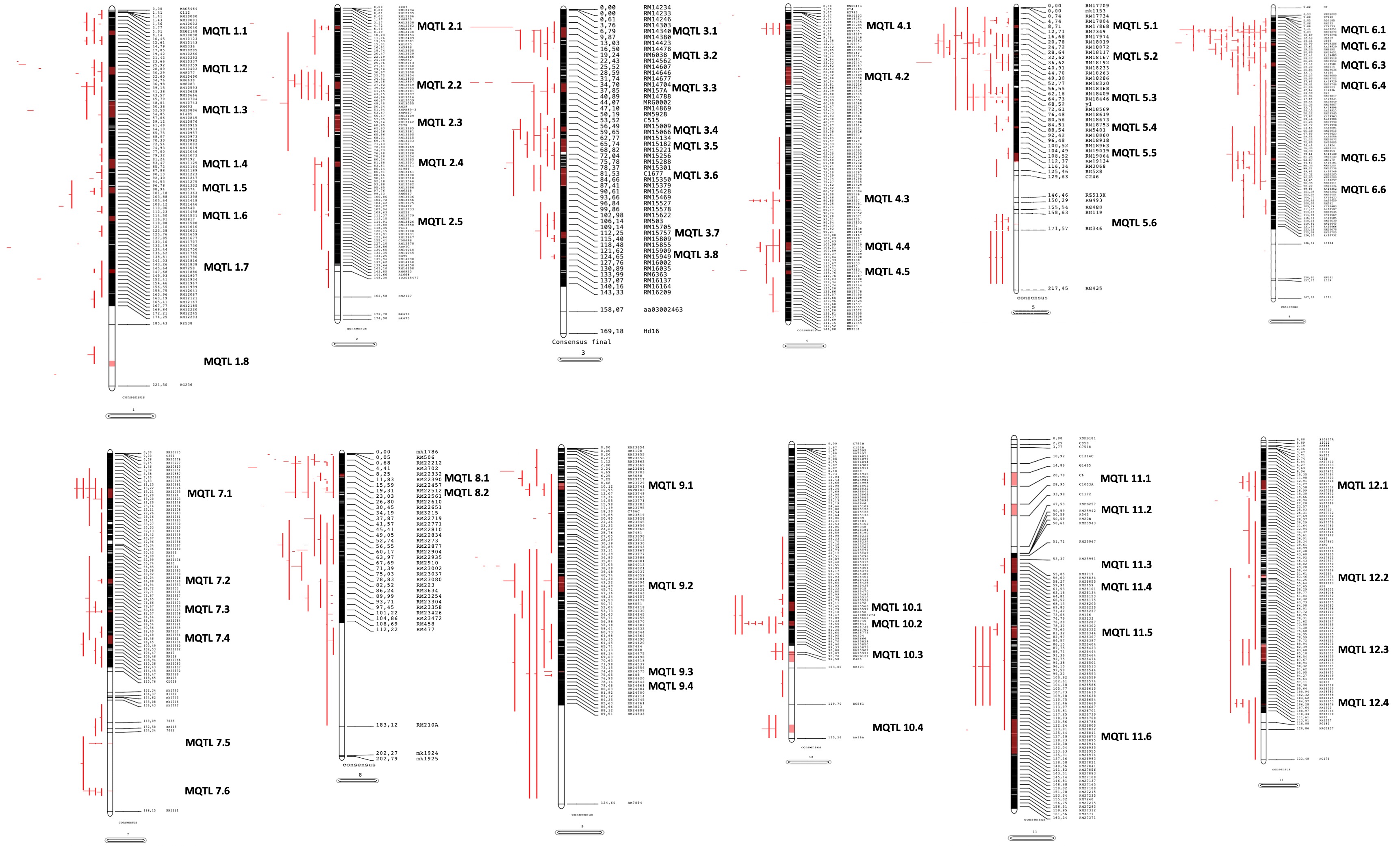

Supplement: Supplementary file 2 [file Image_2.jpg]
